# Tuberculosis in patients with systemic lupus erythematosus

**DOI:** 10.3389/fimmu.2025.1625748

**Published:** 2025-09-01

**Authors:** Jomkwan Ongarj, Porntip Intapiboon, Rachel Tanner, Nawamin Pinpathomrat

**Affiliations:** ^1^ Department of Biomedical Sciences and Biomedical Engineering, Faculty of Medicine, Prince of Songkla University, Songkhla, Thailand; ^2^ Department of Biology, University of Oxford, Oxford, United Kingdom; ^3^ Department of Internal Medicine, Faculty of Medicine, Prince of Songkla University, Songkhla, Thailand

**Keywords:** tuberculosis, systemic lupus erythematosus, immunology, corticosteroids, pathogenesis

## Abstract

Tuberculosis (TB) is an infectious disease caused by *Mycobacterium tuberculosis* (*M. tb*), with approximately 10 million new cases reported worldwide annually. Patients with immunocompromised states or those receiving immunosuppressive therapy for autoimmune diseases are at higher risk of *M. tb* infection or reactivation. The chronic autoimmune disease, systemic lupus erythematosus (SLE), is associated with a higher risk of *M. tb* infection and TB disease during conventional treatment with corticosteroids and immunosuppressants. However, whether risk of TB is influenced by the immune disturbances associated with active SLE when patients are not receiving immunosuppressant treatment remains unclear. In this review, we describe the pathogenesis of TB and SLE and consider how autoimmune responses in SLE could influence TB risk.

## 1 Introduction

Tuberculosis (TB) is an infectious disease caused by *Mycobacterium tuberculosis* (*M. tb*), with an estimated 10.8 million new cases and 1.25 million associated deaths globally in 2023. TB is a major cause of morbidity and mortality (1, 2). It ranked among the top five causes of death in low-income countries and was the 13^th^ leading cause of death globally between 2000 and 2019. Patients with autoimmune diseases, especially those receiving immunosuppressive therapy or in an immunocompromised state, are at particularly high risk (3, 4), and an estimated 161,000 deaths from TB were reported among HIV/AIDS patients in 2023 (5, 6). From 1921 to date, the live-attenuated *M. bovis* Bacillus Calmette-Guérin (BCG) vaccine has been the only TB vaccine licenced for use in humans. The BCG vaccine is effective in preventing severe forms of TB in children. However, the efficacy of BCG in treating pulmonary TB in adults (the most common and transmissible form) is variable (7).

Systemic lupus erythematosus (SLE) is a chronic autoimmune disease that causes inflammation and can damage multiple organs, resulting from loss of tolerance to self-antigens (8). Patients diagnosed with SLE typically require treatment with corticosteroids and immunosuppressive drugs, which places them at a heightened risk of infections (9). Notably, increased rates of *M. tb* infection and TB disease are reported in patients with SLE (10, 11); however, the immunological interplay between the two diseases remains poorly-characterised.

During *M. tb* infection, innate and adaptive immune responses to the pathogen are induced, and cell-mediated immunity is vital in host control. CD4+ T helper (Th) cells proliferate and differentiate into Th1, Th2, and Th17 cells, secreting pro-inflammatory cytokines (among others) that contribute to the ability of mononuclear cells to control *M. tb* infection. In addition, cytotoxic CD8+ T cells are activated to kill infected cells directly through secretion of perforin and granzymes (12, 13) and releasing cytokines such as IFN-γ and TNF-α. This inflammation is crucial in controlling *M. tb* infection, but can also lead to tissue damage and disease progression (14).

Similarly, SLE is characterised by a generalised systemic inflammation, which causes CD4+ T cells to damage self-tissue when activated by self-antigens (15, 16). In particular, Th1 cells are stimulated to produce pro-inflammatory cytokines such as IFN-γ (17). Patients with SLE reportedly have a higher risk of TB, although this is generally thought to be associated with receiving immunosuppressive treatments, with higher doses of the drugs leading to an increased rate of *M. tb* infection and/or reactivation of disease (18–21). Moreover, risk of infection in SLE patients has been reported to associate with dose management (22, 23).

The risk of TB in patients with SLE not undergoing immunosuppressive treatments remains unclear. Considering the benefits of inflammation in controlling *M. tb*, one may hypothesise that inflammatory responses in patients with SLE could confer some degree of protection against *M. tb* infection or TB. For example, mice treated with lipopolysaccharide (LPS) to induce a transient inflammatory environment showed enhanced protection against *M. tb* for up to 6 months post-infection (24). However, a recent *ex vivo* study reported that SLE patients, after short-term treatment, demonstrated better control of mycobacterial growth compared to newly diagnosed patients (25). In other autoimmune states, such as diabetes, additional factors perturbing the immune response of the host may override any positive benefits of inflammation, leading to increased susceptibility (26). Here, we discuss the immunopathogenesis of *M. tb* infection and SLE and consider how they may interact to influence the risk of TB in patients with SLE.

## 2 Methodology

A literature review was conducted to identify studies reporting the characteristics, risks, and incidence of *M. tb* infection and TB disease in SLE patients, as well as BCG vaccination in the context of SLE, using the following approach: PubMed and Web of Science were searched for relevant articles using combinations of the terms Tuberculosis, Systemic Lupus Erythematosus, Incidence, Prevalence, Risk factors, Infection, Vaccination, BCG, Immune response and Cytokines. Inclusion criteria were i) studies reporting original data on TB incidence, prevalence, or risk factors in SLE patients; ii) reports published in peer-reviewed journals; and iii) studies providing sufficient methodological detail for assessment. Exclusion criteria were i) articles written in languages other than English; ii) conference abstracts; and iii) studies lacking primary data on SLE and/or TB.

## 3 Mycobacterium tuberculosis

### 3.1 Characterisation of *M. tb*

*Mycobacterium tuberculosis* causes TB and is believed to have originated 3 million years ago in East Africa. It is believed that bacteria in the genus, *Mycobacterium*, were discovered in the soil, and some species evolved the ability to colonise mammalian hosts (27–29). Robert Koch, a German microbiologist, was the first to isolate the causative agent of TB in 1882 from animal and patient specimens. One year later, it was identified as *M. tb* (30). Over the past 200 years, TB is thought to have claimed over 1 billion lives (31).

The organisms appear as slightly curved rods, 2–4 μm long and 0.2–5 μm wide, and are non-motile and non-sporulating. Other bacteria are commonly stained and identified using standard light microscopy; however, the mycolic acid-rich cell wall of *M. tb* prevents it from absorbing conventional stains, requiring acid-fast staining techniques for visualization. Under ideal conditions, *M. tb* is a slow-growing organism with a doubling period of 12–24 hours. A fundamental feature is its peculiar cell wall structure, providing robust protection against harmful chemicals and therapeutics, and being essential in disease pathogenesis (27, 32).

### 3.2 Immunopathogenesis of *M. tb* infection

**Figure 1:**
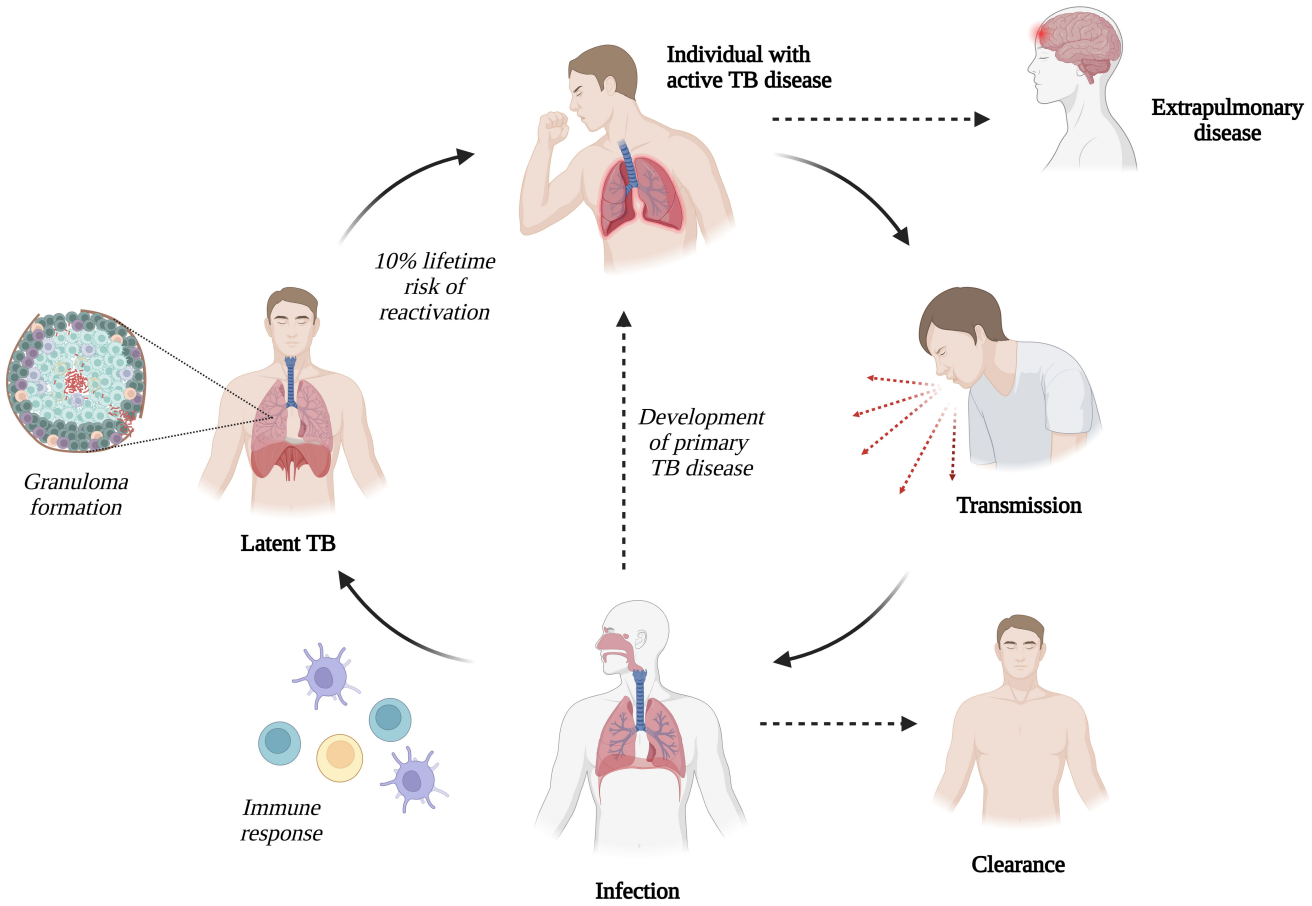
Transmission of *Mycobacterium tuberculosis (M. tb)* infection (Created with BioRender.com).

Following coughing or sneezing by an infected patient, microscopic aerosol droplets containing *M. tb* can remain in the air for several hours and may enter the airways of exposed individuals. After inhaling *M. tb*, one of the following outcomes ensues: [1] no infection, [2] infection with clearance, [3] host control of infection but bacteria remain without symptoms (latent TB infection), or [4] active TB disease (17, 33) (**Figure 1**). Most individuals infected with *M. tb* do not develop active TB disease; however, there is a 10% lifetime risk of reactivation (34).

After inhaling *M. tb*, resident lung alveolar macrophages are the first cells to encounter and phagocytose mycobacteria (35). Similarly, *M. tb* may invade and replicate in alveolar epithelial type II cells (36). Additionally, dendritic cells (DCs) are critical in early infection and can enhance innate and adaptive immune responses. Macrophages and DCs, as professional antigen-presenting cells (APCs), can present *M. tb* antigens to naïve CD4+ and CD8+ T cells on surface MHC class II and MHC class I, respectively (37).

Alveolar macrophages and DCs are crucial in the innate immune response against *M. tb*. Phagocytosis begins when macrophages recognise pathogen-associated molecular patterns through specific pathogen-recognition receptors including Toll-like receptors (TLRs), particularly TLR2 and TLR4, which initiate signaling pathways that activate innate immune responses. This interaction leads to upregulating the transcription of pro-inflammatory cytokines, such as tumour necrosis factor (TNF)-α, interleukin (IL)-1, and IL-12, chemokines, and nitric oxide (38). There are three primary outcomes following the uptake of *M. tb* into macrophages: [1] necrosis, [2] apoptosis, or [3] survival of the infected macrophages, inside which *M. tb* can replicate and potentially infect other cells after macrophage death.

Neutrophils are a source of specific cytokines, which can aid early recruitment and activation of other innate immune cells (39). When DCs present antigens to naïve T-cells, an adaptive response is initiated in the lungs and lymph nodes. The initial activation and proliferation of CD4+ T cells is crucial for the production of IL-2, TNF-α, and interferon (IFN)-γ. IFN-γ is essential for macrophage activation resulting in intracellular killing (17). Pathogen-specific T cells also contribute to granuloma formation (40), where activated macrophages accumulate at the infection site. A crucial feature of granuloma formation is the development of fibrosis within the granuloma and the surrounding lung parenchyma, causing TB lesions (41).

### 3.3 Cell-mediated immune response to *M. tb* infection

The nature of the cellular response is a crucial factor in the development of *M. tb* infection. Following antigen presentation, CD4+ or CD8+ T cells can produce two or more cytokines simultaneously, and such polyfunctional T cells may exert an effect superior to that of single cytokine-producing cells (42). Th1 cells are instrumental in controlling *M. tb* infection by producing IFN-γ, which activates macrophages. IFN-γ induces the transcription of more than 200 genes in the macrophages, including those that encode antimicrobial molecules and induce nitric oxide synthesis, enhancing ability to control bacterial growth (43–45).

Th1 cytokine-producing cells are central to cell-mediated immunity against viral pathogens and intracellular bacteria (46). TNF-α production generally precedes IFN-γ synthesis. During control of mycobacterial infection, TNF-α is likely critical in attracting migrating immune cells to the infection site, contributing to granuloma formation, apoptosis, and controlling disease progression (47). The Th2 response promotes antibody-mediated immunity through the production of cytokines such as IL-4. Th17 cells are critical for immunity against extracellular bacterial and fungal pathogens and participate in the inflammatory response at an early stage of mycobacterial infection. In addition, IL-17 produced by Th17 cells activates polymorphonuclear granulocytes and contributes to lung protective immunity early after vaccination (43, 48).

**Figure 2:**
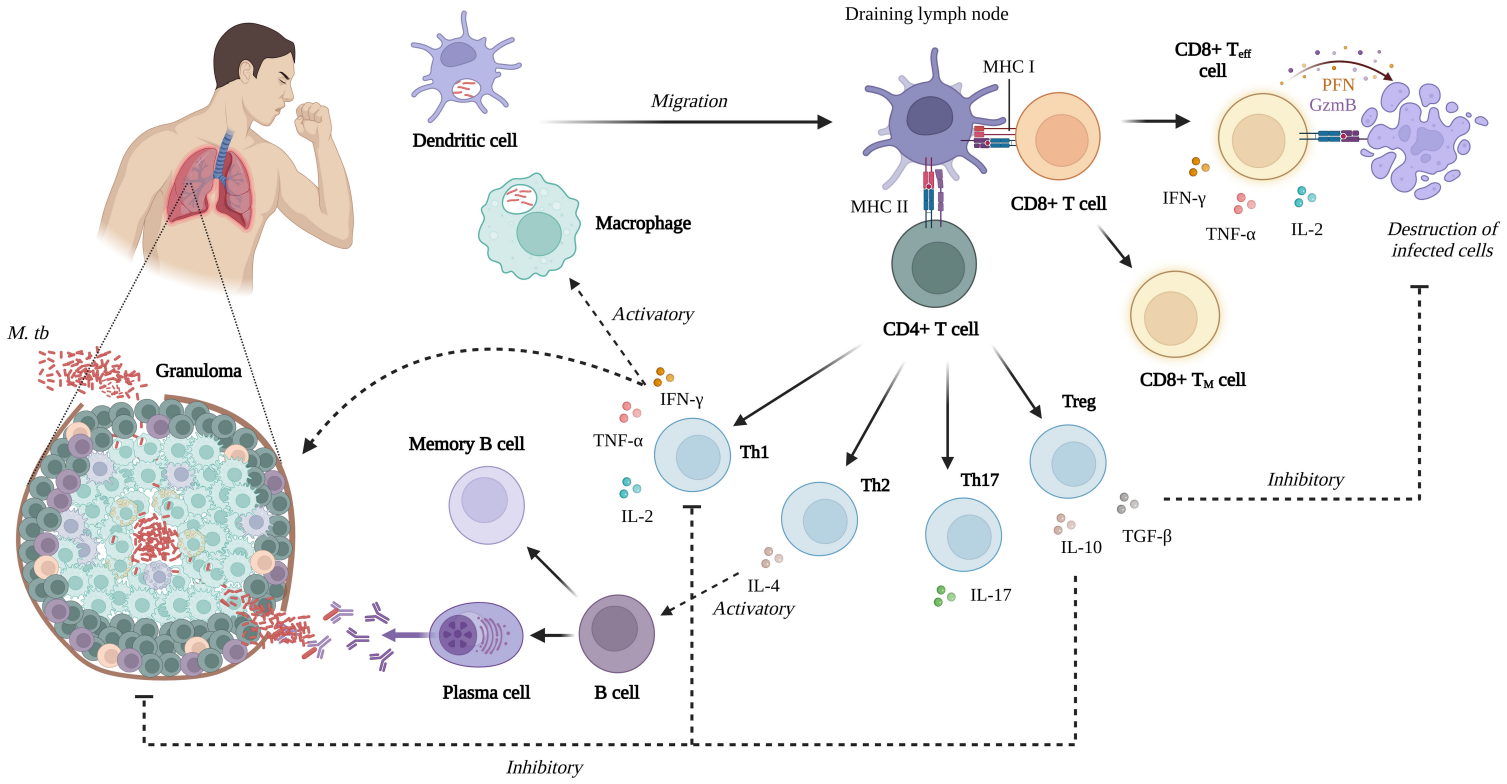
Summary of the cell-mediated immune response of *M. tb* infection (Created with BioRender.com).

During infection, CD8+ or cytolytic T cells are essential in secreting perforin and granulysin, which destroy infected host cells and directly attack *M. tb* (44). Antigen-dependent T cells proliferate rapidly, generating differentiated effector T cells and long-lived memory T cells that spread throughout the body. Memory T cells can mount a fast and robust response to antigens upon re-exposure (49, 50). When the memory response is directed toward the site of pathogen infection, it may work more effectively than the primary response owing to a subpopulation of memory cells known as tissue resident memory cells, which can remain in tissues for lengthy periods without recirculating in the blood and are ready to respond rapidly to a new infection (51) (**Figure 2**).

## 4 Autoimmune disease

### 4.1 Characterisation of autoimmune disease

The human immune system has specific mechanisms for recognising and eliminating foreign antigens to protect against infections. During immune system maturation, central and peripheral tolerance eliminate self-reactive T and B cells (52). The aetiology of self-tolerance loss in autoimmune diseases is complex, involving genetic and environmental factors that lead to ongoing immune activation and tissue damage (53).

**Figure 3:**
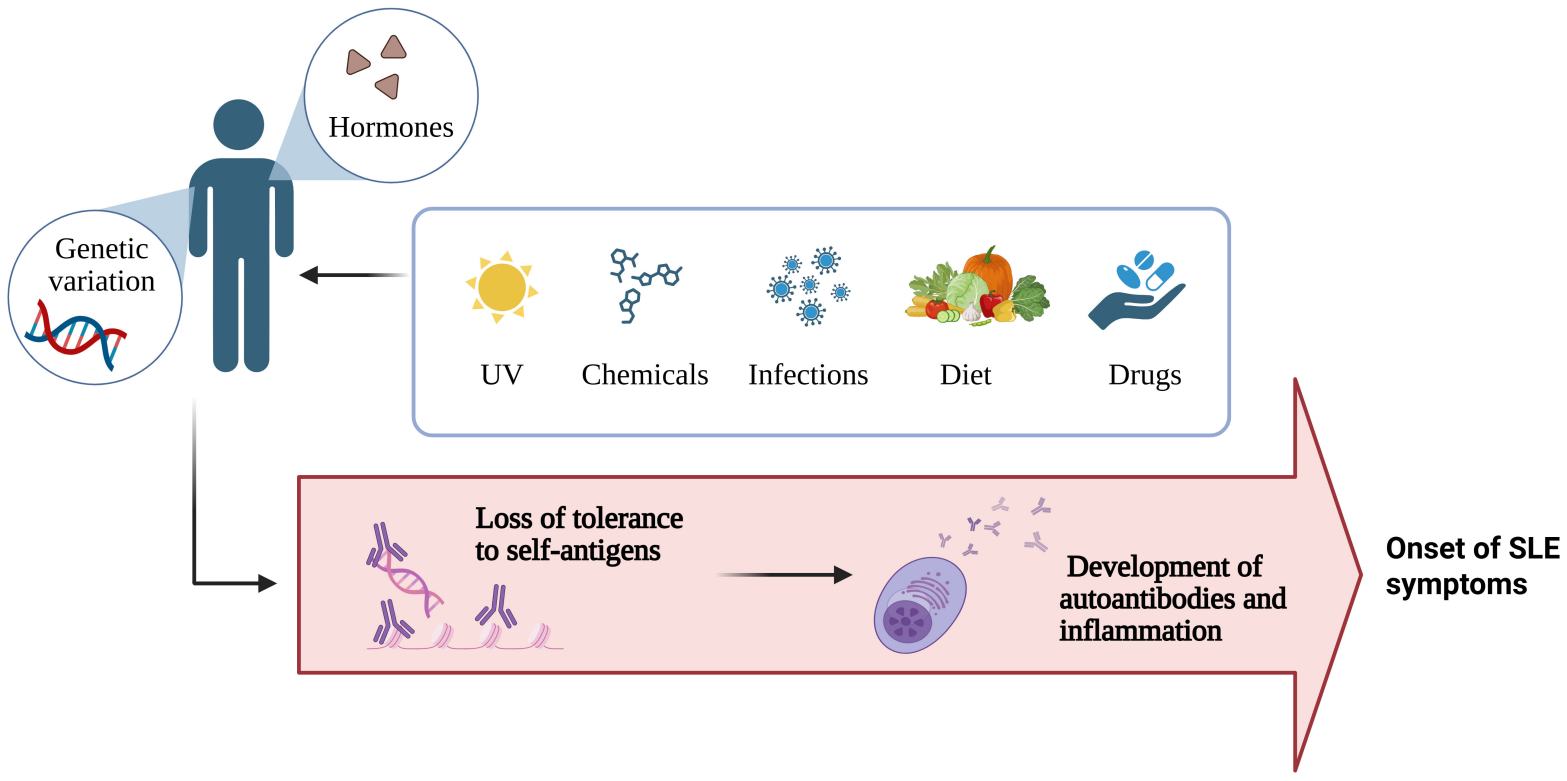
Summary of the factors influencing the development of autoimmune disease (Created with BioRender.com).

In many cases, a family history of autoimmune disease associates with increased risk of disease development compared with the general population. Genetic variations contributing to systemic autoimmune disease comprise three main categories: [1] rare (<1%) genetic polymorphisms and copy-number variants, [2] common (>1%) single-nucleotide polymorphisms and copy-number variants, and [3] epigenetic modifications (54). However, genetic variables account for only one-third of the risk of developing autoimmune diseases, and non-heritable environmental factors account for the remaining ~70% (**Figure 3**). Chemicals, hormones, diets, drugs, and infections may be crucial in determining autoimmune outcomes. Notably, environmental factors can contribute to the development of autoimmune disease in genetically susceptible individuals, and self-tolerance may be overcome in those who are not genetically predisposed (53, 54).

Autoimmune diseases are broadly divided into two types: [1] organ-specific autoimmune diseases, which affect discrete targets, and [2] systemic diseases that involve multiple tissues and organs, including SLE, rheumatoid arthritis (RA), and systemic sclerosis (55).

### 4.2 Immunopathogenesis of SLE

SLE is a chronic autoimmune disease with several immunological abnormalities and clinical manifestations. Although SLE has a significant hereditary component, environmental factors contribute to and catalyse the onset of the disease (56). SLE can affect almost every organ, particularly the kidneys and the central nervous system, with symptoms ranging from mild skin irritation to severe organ damage, primarily driven by abnormal immune function (57, 58).

Autoantibodies against self-nuclear antigens, particularly double-stranded DNA, form immune complexes that trigger inflammation and activate the classical complement pathway to clear apoptotic debris, often causing tissue damage (16, 59). While the complement system generally protects against infection and maintains tissue homeostasis, genetic deficiencies in key components such as C1, C2, and C4 increase susceptibility to SLE and systemic infections (60, 61). For instance, a reported case of an 11-year-old with SLE showed severely reduced complement activity and concurrent *Staphylococcus aureus* bacteraemia (62). The pathogenesis of SLE involves mechanisms like impaired clearance of immune complexes and defective handling of apoptotic debris (63).

**Figure 4:**
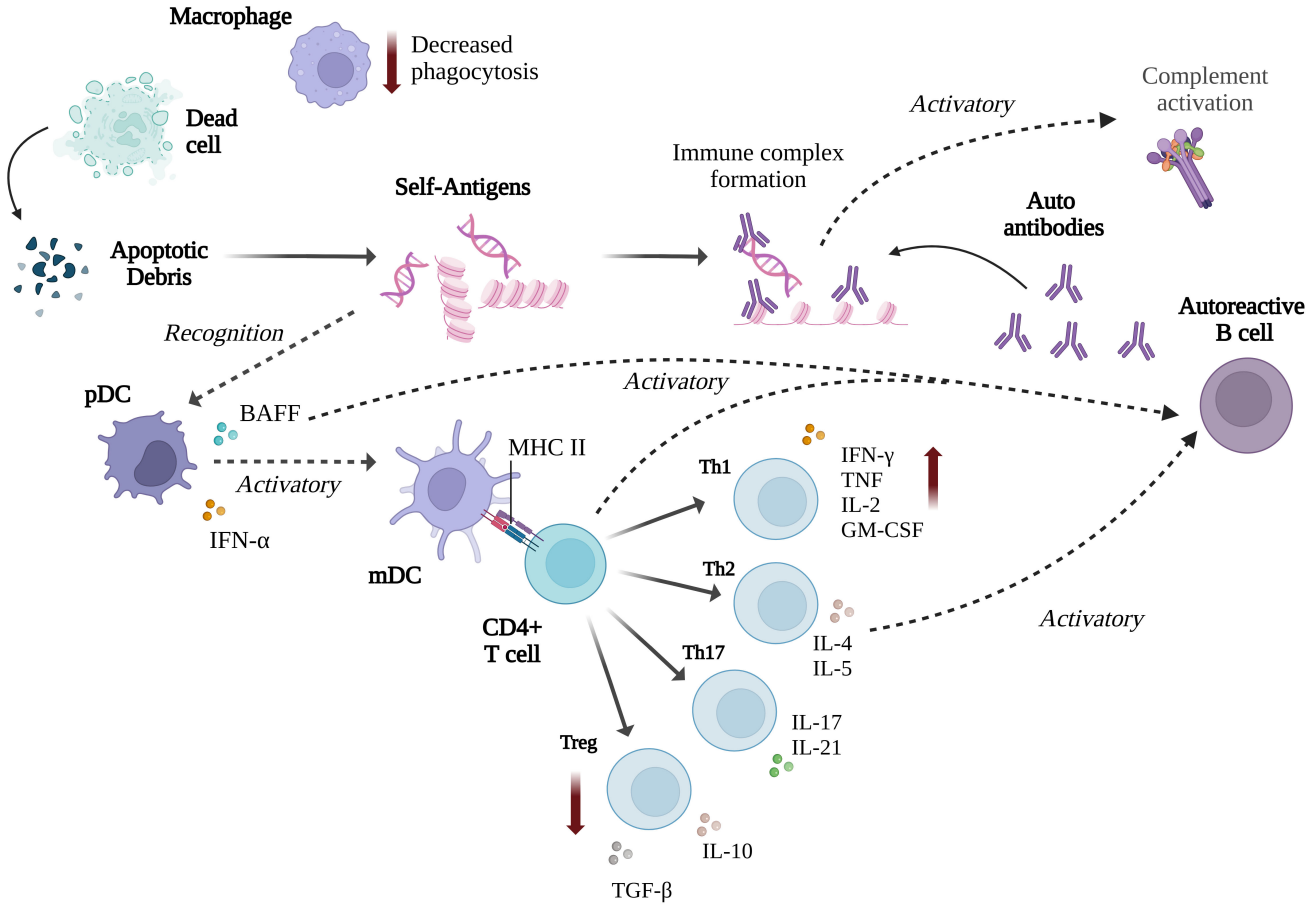
Summary of the immune responses involved in SLE (Created with BioRender.com).

Many environmental and genetic factors can influence the loss of tolerance in B and T cells, including different Th cell subsets such as Th1, Th2, Th17, T follicular helper, and regulatory T (Treg) cells (15). The innate immune system also plays a role by activating the adaptive immune response and sustaining inflammation (64). Impaired clearance of apoptotic cells, a process dependent on the complement system, is a key factor in SLE development, as it leads to the release of autoantigens that trigger immune detection. Macrophages, which are critical in clearing apoptotic debris (65), exhibit altered function in SLE, with an increase in pro-inflammatory classically-activated macrophages (M1) and a decrease in tissue-repair by alternatively-activated macrophage (M2), contributing to disease pathogenesis (66, 67). Dendritic cells (DCs) play a central role in SLE pathogenesis by presenting apoptotic cell debris as self-antigens, leading to hyperactivation of B and T cells (68). Plasmacytoid DCs, activated by immune complexes through TLR7 and TLR9, produce type I interferons like IFN-α, which stimulate myeloid DCs to migrate to inflammatory sites and promote adaptive immune responses by activating effector T and B cells while suppressing regulatory T cells (69, 70) (**Figure 4**).

Assessment of disease activity in patients with SLE is crucial to direct treatment. Several validated activity indices are used to measure disease activity or provide organ-based indices. These include the British Isles Lupus Assessment Group, Systemic Lupus Activity Measure, European Community Lupus Activity Measure, Systemic Lupus Erythematosus Disease Activity Index (SLEDAI), and Systemic Lupus Erythematosus Disease Activity Index 2000 (SLEDAI-2K). SLEDAI-2K is amended to allow for the documentation of continued disease activities, such as an inflammatory rash, alopecia, mucosal ulcers, and proteinuria, which is new, recurring, or persistent (71, 72). In addition, the levels of Th1, Th2, and Th17 cytokines are usually increased in SLE and are elevated in patients with active SLE compared with those with inactive SLE (73).

Patients with SLE exhibit significant immunological dysfunction, rendering them highly susceptible to a wide range of opportunistic infections, including viral, bacterial, fungal, and parasitic pathogens (74, 75). Among viral infections, varicella-zoster virus is the most prevalent, particularly in patients receiving intensive immunosuppressive therapy, often resulting in reactivation of herpes zoster (shingles) (76, 77). Cytomegalovirus (CMV) is also frequently observed and is notable for its ability to mimic lupus flares and further suppress cell-mediated immunity (78, 79); it has been detected in approximately 40% of active SLE patients (76). Elevated antibody levels against CMV and Epstein-Barr virus have also been reported in patients with SLE (80). Additionally, patients are at increased risk of severe herpes simplex virus infections associated with daily oral doses of steroids (81). However, bacterial infections remain the most common, present in approximately 40% of all cases, with *Streptococcus pneumoniae* being the highest incidence (75). *Staphylococcus aureus* and *Escherichia coli* are also commonly implicated with an infection of 14-30% and 5-20% respectively (82–86). The heightened vulnerability to infection in SLE is attributed to both the immunosuppressive effects of treatment and the underlying immune dysregulation inherent to the disease (87).

## 5 *Mycobacterium tuberculosis* infection and TB disease in patients with SLE

Chronic autoimmune diseases and long-term administration of immunosuppressants have been suggested to increase the risk of infectious complications. SLE is associated with a higher risk of TB disease (88), and therefore, active detection of latent infection and treatment of TB in individuals with SLE is essential (89). In India, a retrospective analysis of 146 SLE cases was conducted over 5 years. 17 of these patients had TB disease (prevalence rate, 11.6%). This prevalence was consistent with that of a cohort in Hong Kong comprising 526 patients with SLE. In Taiwan, 21 of 3,179 patients with SLE were infected with *M. tb* (20, 90, 91).

Despite its relatively low prevalence compared with other infectious disease, TB remains responsible for significant morbidity and mortality in patients with SLE. In a tertiary hospital setting, a retrospective cohort study between 2004 and 2011 showed that 2% of SLE patients had TB (17/841) as an underlying disease (88). When SLE patients with *M. tb* infection were compared with those without infection, the infected group received higher doses of glucocorticoids (GC). The cumulative dose of GC was an associated risk factor (92). In the context of high-activity SLE disease, *M. tb* infection was observed in 23 (10%) of 230 patients with SLE, and those with a SLEDAI score > 12 and total intake of prednisolone > 1000 mg had an even higher risk of infection (22, 93). Consistently, a study conducted in a British cohort found that using corticosteroids at doses ≥10 mg/day was associated with a higher risk of *M. tb* infection (23). A study in Colombia identified several factors significantly associated with the development of active TB in SLE patients, including lymphopenia, a cumulative glucocorticoid steroid dose ≥1830 mg over 12 months and treatment with two or more immunosuppressants. These findings underscore the impact of immunosuppressive therapy on TB susceptibility in SLE patients (94).

Accurate diagnosis of TB in patients with SLE is essential, as TB symptoms can mimic SLE flares. There are similarities but also differences in cytokine profiles between the conditions: SLE is marked by elevated type I interferon responses and multiple pro-inflammatory cytokines, whereas TB is dominated by a Th1 cytokine pattern, especially increased IFN-γ and TNF-α (**Table 1**). Patients with SLE have excessive inflammation and cytokine secretion and may be on immunosuppressant therapies, which can affect the results of routine TB diagnostic tests. The tuberculin skin test (TST) remains the standard method for detecting latent TB infection (LTBI) in many endemic settings. However, it has some limitations, including a higher likelihood of false negatives in patients with immunocompromised states and those taking immunosuppressants (95). The IFN-γ release assay (IGRA) is an alternative, *in-vitro* immunodiagnostic method based on detecting IFN-γ produced by T cells following specific *M. tb* antigen stimulation, improving the diagnostic accuracy for LTBI. IGRA specificity is superior to that of conventional TSTs (96).

**Table 1:** Overview of the cytokine profile in patients with SLE and TB.

Cytokine	SLE	TB	Protective/Pathogenic role
IFN-γ	Elevated	Strongly elevated following infection	Essential role in defence against TB; amplifies immune responses and correlates with disease activity in SLE (73, 101)
IFN-α	Elevated	Elevated	Complex and sometimes detrimental role in TB, essential for controlling *M. tb* infection but excessive or dysregulated production associated with worsened disease outcome. Contributes to immune dysregulation, inflammation and autoimmunity in SLE (47, 102)
IL-10	Elevated	Mostly associated with active TB disease	Anti-inflammatory with paradoxical roles; helps to prevent excessive inflammation and tissue damage in TB, but also impairs bacterial clearance by suppressing Th1 functions. Can exhibit pro-autoimmune and immunostimulatory effects in SLE, contributing to both immune regulation and autoantibody production (102, 103)
IL-17	Elevated	Elevated in active TB disease	Important in early response to *M. tb* infection and granuloma formation, but can also contribute to lung inflammation and pathology. A key proinflammatory cytokine in SLE contributing to disease pathogenesis (43, 73)
IL-2	Decreased	Elevated in active TB disease	Elevated in TB patients contributing to T cell proliferation, differentiation and effector function; decreased in SLE due to impaired T cell function (73, 103)

One study using IGRAs found no indeterminate results in patients with SLE (97). However, another study reported a significantly higher number of undetermined IGRA results in patients with SLE, including those taking immunosuppressants and those not on therapy (32.4%), than in patients with other autoimmune diseases (5.7%) or healthy controls (0%). An inconclusive IGRA result was found in 16.9% of patients with SLE in another cohort and was associated with a higher SLE disease activity index score and an increased dose of GCs (98, 99). Although there are several studies in patients with SLE treated with immunosuppressants, the effect of active SLE disease itself on the accuracy of TB diagnosis remains unclear. This makes it difficult to interpret the rates of *M. tb* infection in patients with SLE compared with those in the healthy population, as they may be underestimated, hindering the determination of relative susceptibility. Immunocompetent and immunosuppressed SLE patients may exhibit different outcomes, illustrating the influence of immunosuppressive drugs on test performance (**Table 2**). A combination of both TST and IGRA may be considered in the context of high TB exposure risk, regardless of immunosuppression status.

**Table 2:** IGRA and TST performance in immunocompetent and immunocompromised SLE patients.

Patient groups	TST	IGRA
Immunocompetent SLE	Useful for LTBI screening; often employed before initiating immunosuppressive therapy. False negative risk: Even in the absence of immunosuppression, SLE-related immune dysregulation can reduce response. False positive risk: Prior BCG vaccination or exposure to NTM cause false positives.	Preferred if individual is BCG vaccinated or about to start immunosuppression. False negative risk: Less affected by mild immune dysfunction. False positive risk: Not affected by BCG vaccination or NTM exposure.
Immunocompromised SLE	Sensitivity reduced by steroid and immunosuppressive treatment, which may result in false-negative results. Minimal direct effect of B-cell targeted therapies such as rituximab. Less reliable than IGRA.	Sensitivity reduced by steroid and immunosuppressive treatment, and indeterminate results may be more frequent in patients with high disease activity (95, 104), but more robust than TST. Preferred in those starting immunosuppressants or on current immunosuppressants.

Despite ongoing debate regarding the utility of IGRA in non–anti-TNF settings, international guidelines consistently recommend screening for LTBI before initiating biological or targeted synthetic DMARDs (bDMARDs or tsDMARDs) (100). Notably, anti-TNF agents are not part of the standard therapeutic strategy for SLE, and currently, there is no universally accepted guideline or robust evidence supporting TB prophylaxis specifically in SLE. Nevertheless, to facilitate early detection of LTBI, we emphasise the importance of TB screening with chest radiography or computed tomography (CT) scans for active TB exclusion. In low TB prevalence areas, the use of IGRA is particularly favoured, aligning with expert opinion that supports LTBI screening prior to initiating glucocorticoids and immunosuppressive agents. These recommendations encourage adherence to national and/or international guidelines and generally favour IGRA over the traditional TST.

### 5.1 Risk of *M. tb* infection in patients with new-onset SLE

As discussed, the autoimmune state of SLE is associated with generalised systemic inflammation, particularly elevated Th1 cytokine profiles in patients with active SLE compared to those with inactive SLE or before reaching an immunocompromised state and before treatment with immunosuppressants (73). New diagnosis of *M. tb* infection has been reported with a median of two years after diagnosis with SLE (10, 73). The potential relationship between SLE and altered susceptibility to *M. tb* infection or TB disease may be related to the types of cytokines involved. IFN-γ and TNF-α are secreted at high levels in response to self-antigen in SLE disease and are central to immunity against *M. tb* (73, 105, 106). TNF-α plays a significant role in immune cell recruitment, activation, apoptosis, and differentiation and is a critical pro-inflammatory cytokine governing TB pathogenesis (107, 108). In patients with SLE, TNF-α levels are increased and correlate with disease activity that contributes to the immunopathogenesis of SLE (109, 110).

In addition, Th17 cells are crucial in protecting against extracellular pathogens and mediating inflammatory responses, particularly in autoimmune and chronic inflammatory diseases (13). IL-17 may have evolved to protect the host mucosa from primary infections by intracellular bacteria such as *M. tb* (111). The upregulated secretion of these cytokines and, particularly, the overexpression of systemic inflammatory factors in patients with SLE may be associated with improved protection against *M. tb* infection. In murine models, low-dose LPS was used to generate an increased acute systemic and pulmonary inflammatory response, conferring protection against *M. tb* infection with a reduced *M. tb* burden for the duration of the study (up to 6 months post-infection) compared to non-LPS treated mice. The transient inflammatory environment was associated with a neutrophil and CD11b^+^ cell influx and increased inflammatory cytokines, including TNF-α, IL-1β, and IL-6 (24).

It may be relevant to consider the effects of increased basal inflammation observed with aging, which is characterized by elevated levels of circulating pro-inflammatory cytokines such as TNF and IL-1β (112). Although older adults are known to be more susceptible to TB, the chronic inflammatory status of old mice has been associated with early control of *M. tb* infection compared with younger mice, which may be CD8+ T cell-mediated, facilitated by Th1 cytokines, and associated with the pre-activation of innate cells in the lungs (113–118). However, unlike in LPS-stimulated mice, old mice cannot sustain *M. tb* control, likely owing to reduced adaptive immune function (119), highlighting the potentially contrasting influences of acute compared with chronic inflammation. At the other end of the age spectrum, inflammation and immune activation in South African infants are associated with an increased risk of *M. tb* infection (120) and T-cell activation (likely driven by persistent infections such as CMV) with a risk of TB disease (121, 122).

Our recent study in Southern Thailand compared ability to control mycobacterial growth *ex vivo* using peripheral blood mononuclear cells (PBMC) collected from active SLE patients, SLE patients treated for 3 months or 6 months, patients with inactive SLE, and healthy control groups. Newly-diagnosed active SLE patients prior to treatment showed poor control of mycobacterial growth, and growth control was inversely correlated with SLE disease activity (25). This is consistent with aforementioned epidemiological data supporting a higher risk of TB in SLE patients. It was also noted that SLE patients who had been treated for 6 months had enhanced control of mycobacterial growth compared to healthy controls and those with active SLE (25). Improved ability to control mycobacteria after immunosuppressive treatment may be associated with restoration of the Th1/Th2 balance or may simply reflect a reduced effect of SLE drugs *ex vivo*. Interestingly, this group also had the highest frequencies of CD8+ T cells, NK cells and NKT cells producing IFN-γ and/or TNF-α, and proinflammatory cytokine-producing NK and NKT cells correlated with mycobacterial growth inhibition at the individual patient level. A role for these cell types in controlling mycobacterial growth may inform the development of effective immunotherapeutic strategies to reduce the risk of TB in SLE patients (25).

Infective mycobacteria share antigen homology with the human host, increasing the possibility of a beneficial cross-reactive adaptive immune response. Mycobacterial infections and autoimmune diseases share certain immunopathological features, including molecular mimicry between microbial glycolipids and host DNA. In patients with active TB, autoantibodies such as anti-nuclear antibodies (ANA), rheumatoid factors, and anti-DNA antibodies have been detected. These anti-DNA antibodies may cross-react with glycolipids that are conserved across all strains of mycobacteria (123). This suggests a potential mechanism of cross-reactivity between *M.tb* antigens and self-antigens. A study reported that 60% of 57 patients with active pulmonary TB exhibited elevated levels of the 16/6 anti-DNA idiotype, which is associated with anti-DNA autoantibodies, whereas only 4% of healthy control sera tested positive (124). Monoclonal autoantibodies derived from patients with SLE reportedly recognise mycobacterial antigens, including cell wall-derived glycolipids. In contrast, there is evidence from animal models and arthritis patients that *M. tb*-reactive T cells can recognise self-antigens, and mycobacteria could drive some of the clinical manifestations of SLE (125). In general, autoantibodies appear to be detrimental in terms of TB risk, with significantly-raised ds-DNA antibodies in patients with concurrent SLE and TB compared with SLE-only controls (126), and the prevalence of anti-IFN-α autoantibodies (found in up to 40% of patients with SLE) associated with a higher frequency of *M. tb* infection (127). Although immune dysregulation during chronic SLE and treatment with high doses of corticosteroids/immunosuppressants are associated with increased susceptibility to TB, whether the inflammation observed during active SLE disease before this state is sufficient to confer benefits in enhancing protection against *M. tb* or these are outweighed by other immune perturbations remains unclear.

High-dose corticosteroid therapy further amplifies the risk of TB in SLE by broadly suppressing immune function. It impairs macrophage activation, reduces TNF-α and IFN-γ production, and weakens Th1 responses—key mechanisms needed to contain *M. tb* (128, 129). The prescription pattern of immunosuppressive therapy in SLE typically begins with high-dose corticosteroids for severe disease manifestations or pulse (“megadose”) corticosteroids in cases of organ- or life-threatening involvement. The initiation of other immunosuppressive agents or biologic therapies often follows this. Prolonged exposure to corticosteroids is a well-established independent risk factor for increased susceptibility to infections, including TB. Corticosteroids exert broad immunosuppressive effects by impairing both innate and adaptive immune responses. They inhibit lymphokine production, reduce monocyte chemotaxis, suppress IL-1 and TNF secretion, and impair T-cell activation (130). The magnitude of these immunosuppressive effects correlates with both glucocorticoid dose and treatment duration; however, even a sustained prednisone dose of 7.5 mg/day carries a substantially elevated infection risk. Indeed, it has been shown that for every 10 mg/day increase in prednisone dosage, the risk of experiencing a serious infection increases elevenfold (85).

### 5.2 TB vaccines and SLE

The BCG vaccine against TB is a live-replicating vaccine that is primarily administered at birth in countries with a high TB burden and is generally not recommended for patients with SLE (131). If administration of live attenuated vaccines such as BCG is being considered in individuals with potential immunosuppression, it should be given prior to the initiation of immunosuppressive therapy; for example live-attenuated varicella zoster virus (VZV) vaccine should be administrated at least four weeks prior to immunosuppressive treatment (132). Once immunosuppression begins, BCG is contraindicated due to risk of disseminated BCG infection (BCGosis). Hence, the potential benefits of protection against TB should be carefully weighed against the risk of vaccine-related adverse events.

BCG may be a double-edged sword in autoimmune diseases, with evidence of inducing autoimmune conditions and potential protective effects (133). As previously described, there is some cross-reactivity between mycobacterial and human antigens, and autoantibodies are identified in patients infected by mycobacteria. IgG responses at 8 weeks post-BCG vaccination exhibit increased reactivity to host ion transporters, cytokine receptors, other cell surface receptors, ribonucleoproteins, and enzymes (134). This modification of the host immunological and non-immunological landscapes may influence responses to TB and autoimmune diseases.

In non-obese diabetic mice, heat-killed BCG prevented diabetes but precipitated an SLE-like syndrome (135). Although rare, BCG administration, particularly intravesically for treating bladder cancer, can trigger autoimmune phenomena or full-blown autoimmune diseases including lupus vulgaris (136). However, evidence of the therapeutic effects of BCG is observed in at least two human autoimmune diseases, multiple sclerosis and type 1 diabetes, likely associated with increased Treg function in controlling inflammation (137–139). Preliminary evidence suggests there could have been a similar beneficial role of BCG in improving SLE in the MRL/LPR mouse model and a limited number of patients with SLE (140, 141); however, further studies are required.

Due to the insufficiency of BCG in protecting against pulmonary disease in TB endemic regions, a robust pipeline of new vaccines for TB are under development. M72/AS01E vaccine is a protein subunit vaccine that has been shown to provide 49.7% efficacy against progression to TB disease in adults with latent TB infection in phase IIb trials (142). Moreover, in early-phase trials, the live-attenuated vaccine candidate MTBVAC induced similar or superior immune responses compared to BCG (143). TB vaccine trials to date have largely focused on healthy adults or those with latent TB infection and excluded participants with autoimmune conditions or those using immunosuppressive drugs. However, M72/AS01 and VPM1002 (a recombinant BCG vaccine) have been shown to be safe in HIV-positive individuals and HIV-exposed infants respectively (144). Early phase studies could eventually be considered in stable autoimmune patients, as TB vaccines must be proven safe for use in vulnerable populations including autoimmune cohorts in high-TB burden settings and those on biologics. Inactivated or subunit vaccines may be more suitable in this context, and precedent includes a study conducted in female SLE patients receiving the quadrivalent human papillomavirus (HPV) vaccine which demonstrated that the vaccine was safe, well tolerated, and not associated with any exacerbation of disease activity (145). Until robust studies confirm the safety of vaccine administration in individuals with autoimmune disorders, a cautious approach is advised, and vaccination in SLE patients should be carefully evaluated on a case-by-case basis, taking into account disease stability and time relative to immunosuppressive therapy.

## 6 Conclusion

Patients with SLE have a higher risk of *M. tb* infection and TB disease and higher associated mortality rates than healthy individuals. A primary contributing factor is immunosuppressive therapy, particularly conventional treatment with high doses of corticosteroids administered to patients with SLE during periods of high disease activity. The systemic inflammation observed in patients with SLE comprises an increased immune response against their tissues, with a particular increase in Th1-secreted pro-inflammatory cytokines, which are critical in controlling *M. tb*. Thus, better understanding how immune responses affect the risk of TB in SLE remains essential. These findings could have implications for managing patients with SLE and identifying correlates of protective immunity against TB, which may direct the rational design of improved vaccines and therapeutics.

## References

[1] Organization WH. Global tuberculosis report 2023. Geneva, Switzerland: World Health Organization (2023). Available online at: https://www.who.int/publications/i/item/9789240083851 (Accessed November 7, 2023).

[2] Bagcchi S. WHO’s global tuberculosis report 2022. Lancet Microbe. (2023) 4:e20. doi: 10.1016/S2666-5247(22)00359-7, PMID: 36521512

[3] McGrath B Broadhurst M Roman C. Infectious disease considerations in immunocompromised patients. JAAPA. (2020) 33:16–25. doi: 10.1097/01.JAA.0000694948.01963.f4, PMID: 32841972

[4] Thomas K Vassilopoulos D. Immunization in patients with inflammatory rheumatic diseases. Best Pract Res Clin Rheumatol. (2016) 30:946–63. doi: 10.1016/j.berh.2016.10.009, PMID: 27964798

[5] Organization WH. WHO reveals leading causes of death and disability worldwide: 2000–2019, Geneva, Switzerland: World Health Organization. Vol. 21 (2020). 2021.

[6] Organization WH. Global tuberculosis report 2021. In: License: CC BY-NC-SA 3.0 IGO. World Health Organization, Geneva (2021). Available online at: https://www.who.int/publications/i/item/9789240037021 (Accessed October 14, 2021).

[7] du Preez K Seddon JA Schaaf HS Hesseling AC Starke JR Osman M. Global shortages of BCG vaccine and tuberculous meningitis in children. Lancet Glob Health. (2019) 7:e28–e9. doi: 10.1016/S2214-109X(18)30474-1, PMID: 30554756

[8] Zharkova O Celhar T Cravens PD Satterthwaite AB Fairhurst AM Davis LS. Pathways leading to an immunological disease: systemic lupus erythematosus. Rheumatol (Oxford). (2017) 56:i55–66. doi: 10.1093/rheumatology/kew427, PMID: 28375453 PMC5410978

[9] Yuan Q Xing X Lu Z Li X eds. Clinical characteristics and risk factors of infection in patients with systemic lupus erythematosus: a systematic review and meta-analysis of observational studies. In: Seminars in Arthritis and Rheumatism. Amsterdam, Netherlands: Elsevier., PMID: 10.1016/j.semarthrit.2020.06.004 32911280

[10] Hamijoyo L Sahiratmadja E Ghassani NG Darmawan G Susandi E van Crevel R. Tuberculosis among patients with systemic lupus erythematosus in Indonesia: A cohort study. Open Forum Infect Dis. (2022) 9. doi: 10.1093/ofid/ofac201, PMID: 35794932 PMC9251660

[11] Xiao X Da G Xie X Liu X Zhang L Zhou B. Tuberculosis in patients with systemic lupus erythematosus–a 37-year longitudinal survey-based study. J Internal Med. (2021) 290:101–15. doi: 10.1111/joim.13218, PMID: 33259665

[12] Kaufmann SHE. Protection against tuberculosis: cytokines, T cells, and macrophages. Ann Rheum Dis. (2002) 61:ii54–ii8. doi: 10.1136/ard.61.suppl_2.ii54, PMID: 12379623 PMC1766701

[13] Lyadova IV Panteleev AV. Th1 and th17 cells in tuberculosis: protection, pathology, and biomarkers. Mediators Inflamm. (2015) 2015:854507. doi: 10.1155/2015/854507, PMID: 26640327 PMC4657112

[14] Sasindran SJ Torrelles JB. Mycobacterium tuberculosis infection and inflammation: what is beneficial for the host and for the bacterium? Front Microbiol. (2011) 2:2. doi: 10.3389/fmicb.2011.00002, PMID: 21687401 PMC3109289

[15] Choi J Kim ST Craft J. The pathogenesis of systemic lupus erythematosus-an update. Curr Opin Immunol. (2012) 24:651–7. doi: 10.1016/j.coi.2012.10.004, PMID: 23131610 PMC3508331

[16] Tsokos GC Lo MS Reis PC Sullivan KE. New insights into the immunopathogenesis of systemic lupus erythematosus. Nat Rev Rheumatol. (2016) 12:716–30. doi: 10.1038/nrrheum.2016.186, PMID: 27872476

[17] Saenz B Hernandez-Pando R Fragoso G Bottasso O Cardenas G. The dual face of central nervous system tuberculosis: a new Janus Bifrons? Tuberc (Edinb). (2013) 93:130–5. doi: 10.1016/j.tube.2012.11.011, PMID: 23305698

[18] Balbi GGM MaChado-Ribeiro F Marques CDL Signorelli F Levy RA. The interplay between tuberculosis and systemic lupus erythematosus. Curr Opin Rheumatol. (2018) 30:395–402. doi: 10.1097/BOR.0000000000000493, PMID: 29438163

[19] Sayarlioglu M Inanc M Kamali S Cefle A Karaman O Gul A. Tuberculosis in Turkish patients with systemic lupus erythematosus: increased frequency of extrapulmonary localization. Lupus. (2004) 13:274–8. doi: 10.1191/0961203303lu529xx, PMID: 15176665

[20] Tam L-S Li EK Wong S-M Szeto C-C. Risk factors and clinical features for tuberculosis among patients with systemic lupus erythematosus in Hong Kong. Scand J Rheumatol. (2002) 31:296–300. doi: 10.1080/030097402760375205, PMID: 12455821

[21] Yun J Lee S Kim T Jun J Jung S Bae S. The incidence and clinical characteristics of Mycobacterium tuberculosis infection among systemic lupus erythematosus and rheumatoid arthritis patients in Korea. Clin Exp Rheumatol. (2002) 20:127–32., PMID: 12051389

[22] Damara I Ariane A Winston K. Predisposing factors of tuberculosis infection in systemic lupus erythematosus patients: A single-center case-control study. Cureus. (2022) 14:e26410. doi: 10.7759/cureus.26410, PMID: 35915698 PMC9337775

[23] Tejera Segura B Rua-Figueroa I Pego-Reigosa JM Del Campo V Wincup C Isenberg D. Can we validate a clinical score to predict the risk of severe infection in patients with systemic lupus erythematosus? A longitudinal retrospective study in a British Cohort. BMJ Open. (2019) 9:e028697. doi: 10.1136/bmjopen-2018-028697, PMID: 31203250 PMC6589043

[24] Piergallini TJ Scordo JM Pino PA Schlesinger LS Torrelles JB Turner J. Acute Inflammation Confers Enhanced Protection against Mycobacterium tuberculosis Infection in Mice. Microbiol Spectr. (2021) 9:e0001621. doi: 10.1128/Spectrum.00016-21, PMID: 34232086 PMC8552513

[25] Ongarj J Intapiboon P Surasombatpattana S Satti I Harris SA Morrison H. Evaluation of immune profiles associated with control of mycobacterial growth in systemic lupus erythematosus (SLE) patients. Tuberculosis. (2024) 148:102533. doi: 10.1016/j.tube.2024.102533, PMID: 38878478

[26] Podell BK Ackart DF Obregon-Henao A Eck SP Henao-Tamayo M Richardson M. Increased severity of tuberculosis in Guinea pigs with type 2 diabetes: A model of diabetes-tuberculosis comorbidity. Am J Pathol. (2014) 184:1104–18. doi: 10.1016/j.ajpath.2013.12.015, PMID: 24492198 PMC3969993

[27] Sakamoto K. The pathology of Mycobacterium tuberculosis infection. Vet Pathol. (2012) 49:423–39. doi: 10.1177/0300985811429313, PMID: 22262351

[28] Gutierrez MC Brisse S Brosch R Fabre M Omaïs B Marmiesse M. Ancient origin and gene mosaicism of the progenitor of Mycobacterium tuberculosis. PLoS Pathog. (2005) 1:e5. doi: 10.1371/journal.ppat.0010005, PMID: 16201017 PMC1238740

[29] Stead WW. The origin and erratic global spread of tuberculosis. How the past explains the present and is the key to the future. Clin Chest Med. (1997) 18:65–77. doi: 10.1016/S0272-5231(05)70356-7, PMID: 9098611

[30] Cambau E Drancourt M. Steps towards the discovery of Mycobacterium tuberculosis by Robert Koch, 1882. Clin Microbiol Infect. (2014) 20:196–201. doi: 10.1111/1469-0691.12555, PMID: 24450600

[31] Paulson T. Epidemiology: A mortal foe. Nature. (2013) 502:S2–3. doi: 10.1038/502S2a, PMID: 24108078

[32] Delogu G Sali M Fadda G. The biology of mycobacterium tuberculosis infection. Mediterr J Hematol Infect Dis. (2013) 5:e2013070. doi: 10.4084/mjhid.2013.070, PMID: 24363885 PMC3867229

[33] Bellini C Horvati K. Recent advances in the development of protein- and peptide-based subunit vaccines against tuberculosis. Cells. (2020) 9. doi: 10.3390/cells9122673, PMID: 33333744 PMC7765234

[34] Rook GAW Dheda K Zumla A. Immune responses to tuberculosis in developing countries: implications for new vaccines. Nat Rev Immunol. (2005) 5:661–7. doi: 10.1038/nri1666, PMID: 16056257

[35] Woo M Wood C Kwon D Park K-HP Fejer G Delorme V. Mycobacterium tuberculosis infection and innate responses in a new model of lung alveolar macrophages. Front Immunol. (2018) 9. doi: 10.3389/fimmu.2018.00438, PMID: 29593716 PMC5858468

[36] Scordo JM Knoell DL Torrelles JB. Alveolar epithelial cells in mycobacterium tuberculosis infection: active players or innocent bystanders? J Innate Immun. (2016) 8:3–14. doi: 10.1159/000439275, PMID: 26384325 PMC4724319

[37] Mihret A. The role of dendritic cells in Mycobacterium tuberculosis infection. Virulence. (2012) 3:654–9. doi: 10.4161/viru.22586, PMID: 23154283 PMC3545947

[38] Ahmad S. Pathogenesis, immunology, and diagnosis of latent Mycobacterium tuberculosis infection. Clin Dev Immunol. (2011) 2011:814943. doi: 10.1155/2011/814943, PMID: 21234341 PMC3017943

[39] Barrios-Payan J Aguilar-Leon D Lascurain-Ledezma R Hernandez-Pando R. Neutrophil participation in early control and immune activation during experimental pulmonary tuberculosis. Gac Med Mex. (2006) 142:273–81., PMID: 17022301

[40] Ehlers S Schaible UE. The granuloma in tuberculosis: dynamics of a host-pathogen collusion. Front Immunol. (2012) 3:411. doi: 10.3389/fimmu.2012.00411, PMID: 23308075 PMC3538277

[41] North RJ Jung YJ. Immunity to tuberculosis. Annu Rev Immunol. (2004) 22:599–623. doi: 10.1146/annurev.immunol.22.012703.104635, PMID: 15032590

[42] Seder RA Darrah PA Roederer M. T-cell quality in memory and protection: implications for vaccine design. Nat Rev Immunol. (2008) 8:247–58. doi: 10.1038/nri2274, PMID: 18323851

[43] Cooper AM. Cell-mediated immune responses in tuberculosis. Annu Rev Immunol. (2009) 27:393–422. doi: 10.1146/annurev.immunol.021908.132703, PMID: 19302046 PMC4298253

[44] Kaufmann SH Hussey G Lambert PH. New vaccines for tuberculosis. Lancet. (2010) 375:2110–9. doi: 10.1016/S0140-6736(10)60393-5, PMID: 20488515

[45] Lin PL Flynn JL. Understanding latent tuberculosis: A moving target. J Immunol. (2010) 185:15–22. doi: 10.4049/jimmunol.0903856, PMID: 20562268 PMC3311959

[46] Kaiko GE Horvat JC Beagley KW Hansbro PM. Immunological decision-making: how does the immune system decide to mount a helper T-cell response? Immunology. (2008) 123:326–38. doi: 10.1111/j.1365-2567.2007.02719.x, PMID: 17983439 PMC2433332

[47] Mohan VP Scanga CA Yu K Scott HM Tanaka KE Tsang E. Effects of tumor necrosis factor alpha on host immune response in chronic persistent tuberculosis: possible role for limiting pathology. Infect Immun. (2001) 69:1847–55. doi: 10.1128/IAI.69.3.1847-1855.2001, PMID: 11179363 PMC98092

[48] Khader SA Bell GK Pearl JE Fountain JJ Rangel-Moreno J Cilley GE. IL-23 and IL-17 in the establishment of protective pulmonary CD4+ T cell responses after vaccination and during Mycobacterium tuberculosis challenge. Nat Immunol. (2007) 8:369–77. doi: 10.1038/ni1449, PMID: 17351619

[49] Cauley LS Lefrancois L. Guarding the perimeter: protection of the mucosa by tissue-resident memory T cells. Mucosal Immunol. (2013) 6:14–23. doi: 10.1038/mi.2012.96, PMID: 23131785 PMC4034055

[50] Mueller SN Gebhardt T Carbone FR Heath WR. Memory T cell subsets, migration patterns, and tissue residence. Annu Rev Immunol. (2013) 31:137–61. doi: 10.1146/annurev-immunol-032712-095954, PMID: 23215646

[51] Sakai S Kauffman KD Schenkel JM McBerry CC Mayer-Barber KD Masopust D. Cutting edge: control of Mycobacterium tuberculosis infection by a subset of lung parenchyma-homing CD4 T cells. J Immunol. (2014) 192:2965–9. doi: 10.4049/jimmunol.1400019, PMID: 24591367 PMC4010124

[52] Bluestone JA. Mechanisms of tolerance. Immunol Rev. (2011) 241:5–19. doi: 10.1111/j.1600-065X.2011.01019.x, PMID: 21488886

[53] Fairweather D. Autoimmune disease: mechanisms. Els. (2007). doi: 10.1002/9780470015902.a0020193.pub2

[54] Wahren-Herlenius M Dörner T. Immunopathogenic mechanisms of systemic autoimmune disease. Lancet. (2013) 382:819–31. doi: 10.1016/S0140-6736(13)60954-X, PMID: 23993191

[55] Fridkis-Hareli M. Immunogenetic mechanisms for the coexistence of organ-specific and systemic autoimmune diseases. J Autoimmune Dis. (2008) 5:1. doi: 10.1186/1740-2557-5-1, PMID: 18275618 PMC2265707

[56] Mak A Tay SH. Environmental factors, toxicants and systemic lupus erythematosus. Int J Mol Sci. (2014) 15:16043–56. doi: 10.3390/ijms150916043, PMID: 25216337 PMC4200809

[57] Moulton VR Tsokos GC. T cell signaling abnormalities contribute to aberrant immune cell function and autoimmunity. J Clin Invest. (2015) 125:2220–7. doi: 10.1172/JCI78087, PMID: 25961450 PMC4497749

[58] Tsokos GC Lo MS Costa Reis P Sullivan KE. New insights into the immunopathogenesis of systemic lupus erythematosus. Nat Rev Rheumatol. (2016) 12:716–30. doi: 10.1038/nrrheum.2016.186, PMID: 27872476

[59] Trouw LA Pickering MC Blom AM. The complement system as a potential therapeutic target in rheumatic disease. Nat Rev Rheumatol. (2017) 13:538–47. doi: 10.1038/nrrheum.2017.125, PMID: 28794515

[60] Ricklin D Hajishengallis G Yang K Lambris JD. Complement: a key system for immune surveillance and homeostasis. Nat Immunol. (2010) 11:785–97. doi: 10.1038/ni.1923, PMID: 20720586 PMC2924908

[61] Skattum L van Deuren M van der Poll T Truedsson L. Complement deficiency states and associated infections. Mol Immunol. (2011) 48:1643–55. doi: 10.1016/j.molimm.2011.05.001, PMID: 21624663

[62] Hagen JP Muller PC Bredius RG ten Cate R. Low complement levels in paediatric systemic lupus erythematosus and the risk of bacteraemia. BMJ Case Rep. (2013) 2013. doi: 10.1136/bcr-2013-010378, PMID: 23966458 PMC3762216

[63] Truedsson L Bengtsson AA Sturfelt G. Complement deficiencies and systemic lupus erythematosus. Autoimmunity. (2007) 40:560–6. doi: 10.1080/08916930701510673, PMID: 18075790

[64] Dema B Charles N. Advances in mechanisms of systemic lupus erythematosus. Discov Med. (2014) 17:247–55., PMID: 24882716

[65] Bijl M Reefman E Horst G Limburg PC Kallenberg CG. Reduced uptake of apoptotic cells by macrophages in systemic lupus erythematosus: correlates with decreased serum levels of complement. Ann Rheum Dis. (2006) 65:57–63. doi: 10.1136/ard.2005.035733, PMID: 15919679 PMC1797975

[66] Mantovani A Sica A Sozzani S Allavena P Vecchi A Locati M. The chemokine system in diverse forms of macrophage activation and polarization. Trends Immunol. (2004) 25:677–86. doi: 10.1016/j.it.2004.09.015, PMID: 15530839

[67] Labonte AC Kegerreis B Geraci NS Bachali P Madamanchi S Robl R. Identification of alterations in macrophage activation associated with disease activity in systemic lupus erythematosus. PLoS One. (2018) 13:e0208132. doi: 10.1371/journal.pone.0208132, PMID: 30562343 PMC6298676

[68] Shao W-H Cohen PL. Disturbances of apoptotic cell clearance in systemic lupus erythematosus. Arthritis Res Ther. (2011) 13:1–7. doi: 10.1186/ar3206, PMID: 21371352 PMC3157636

[69] Blanco P Palucka AK Gill M Pascual V Banchereau J. Induction of dendritic cell differentiation by IFN-α in systemic lupus erythematosus. Science. (2001) 294:1540–3. doi: 10.1126/science.1064890, PMID: 11711679

[70] Jacquemin C Blanco P. The role of dendritic cells in systemic lupus erythematosus. In: Systemic Lupus Erythematosus. Boston, MA, USA: Elsevier (2016). p. 131–6.

[71] Fernando MMA Isenberg DA. How to monitor SLE in routine clinical practice. Ann Rheum Dis. (2005) 64:524–7. doi: 10.1136/ard.2003.015248, PMID: 15769911 PMC1755465

[72] Parker B Bruce IN. 49 - clinical markers, metrics, indices, and clinical trials. In: Wallace DJ Hahn BH, editors. Dubois’ Lupus Erythematosus and Related Syndromes (Ninth Edition). Elsevier, London (2019). p. 614–30.

[73] Muhammad Yusoff F Wong KK Mohd Redzwan N. Th1, Th2, and Th17 cytokines in systemic lupus erythematosus. Autoimmunity. (2020) 53:8–20. doi: 10.1080/08916934.2019.1693545, PMID: 31771364

[74] Jung JY Suh CH. Infection in systemic lupus erythematosus, similarities, and differences with lupus flare. Korean J Intern Med. (2017) 32:429–38. doi: 10.3904/kjim.2016.234, PMID: 28490724 PMC5432804

[75] Dorgham DA Anwar S Khaled AS. Infection in systemic lupus erythematosus patients. Egyptian Rheumatol. (2021) 43:115–8. doi: 10.1016/j.ejr.2020.12.007

[76] Reis AD Mudinutti C de Freitas Peigo M Leon LL Costallat LTL Rossi CL. Active human herpesvirus infections in adults with systemic lupus erythematosus and correlation with the SLEDAI score. Adv Rheumatol. (2020) 60:42. doi: 10.1186/s42358-020-00144-6, PMID: 32831149

[77] Sun F Chen Y Wu W Guo L Xu W Chen J. Varicella zoster virus infections increase the risk of disease flares in patients with SLE: a matched cohort study. Lupus Sci Med. (2019) 6:e000339. doi: 10.1136/lupus-2019-000339, PMID: 31413853 PMC6667776

[78] Ramos-Casals M Cuadrado MJ Alba P Sanna G Brito-Zerón P Bertolaccini L. Acute viral infections in patients with systemic lupus erythematosus: description of 23 cases and review of the literature. Med (Baltimore). (2008) 87:311–8. doi: 10.1097/MD.0b013e31818ec711, PMID: 19011502

[79] Cunha BA Gouzhva O Nausheen S. Severe cytomegalovirus (CMV) community-acquired pneumonia (CAP) precipitating a systemic lupus erythematosus (SLE) flare. Heart Lung. (2009) 38:249–52. doi: 10.1016/j.hrtlng.2008.07.001, PMID: 19486795

[80] Rasmussen NS Draborg AH Nielsen CT Jacobsen S Houen G. Antibodies to early EBV, CMV, and HHV6 antigens in systemic lupus erythematosus patients. Scand J Rheumatol. (2015) 44:143–9. doi: 10.3109/03009742.2014.973061, PMID: 25562120 PMC4389709

[81] Li T-H Lai C-C Wang W-H Chen W-S Tsao Y-P Tsai C-Y. Risk of severe herpes simplex virus infection in systemic lupus erythematosus: analysis of epidemiology and risk factors analysis in Taiwan. Ann Rheum Dis. (2019) 78:941–6. doi: 10.1136/annrheumdis-2018-214844, PMID: 30954968

[82] Al-Rayes H Al-Swailem R Arfin M Sobki S Rizvi S Tariq M. Lupus Around the World: Systemic lupus erythematosus and infections: a retrospective study in Saudis. Lupus. (2007) 16:755–63. doi: 10.1177/0961203307079943, PMID: 17728372

[83] Barrera-Vargas A Gómez-Martín D Merayo-Chalico J Ponce-de-León A Alcocer-Varela J. Risk factors for drug-resistant bloodstream infections in patients with systemic lupus erythematosus. J Rheumatol. (2014) 41:1311–6. doi: 10.3899/jrheum.131261, PMID: 24882843

[84] Dubula T Mody GM. Spectrum of infections and outcome among hospitalized South Africans with systemic lupus erythematosus. Clin Rheumatol. (2015) 34:479–88. doi: 10.1007/s10067-014-2847-0, PMID: 25535200

[85] Ruiz-Irastorza G Olivares N Ruiz-Arruza I Martinez-Berriotxoa A Egurbide M-V Aguirre C. Predictors of major infections in systemic lupus erythematosus. Arthritis Res Ther. (2009) 11:1–8. doi: 10.1186/ar2764, PMID: 19604357 PMC2745791

[86] Tsai PH Jang SS Liou LB. Septicaemia is associated with increased disease activity and mortality in systemic lupus erythematosus: a retrospective analysis from Taiwan. Lupus. (2020) 29:191–8. doi: 10.1177/0961203319899162, PMID: 31959041

[87] Battaglia M Garrett-Sinha LA. Bacterial infections in lupus: Roles in promoting immune activation and in pathogenesis of the disease. J Trans Autoimmun. (2021) 4:100078. doi: 10.1016/j.jtauto.2020.100078, PMID: 33490939 PMC7804979

[88] Yang Y Thumboo J Tan BH Tan TT Fong CHJ Ng HS. The risk of tuberculosis in SLE patients from an Asian tertiary hospital. Rheumatol Int. (2017) 37:1027–33. doi: 10.1007/s00296-017-3696-3, PMID: 28286903

[89] Ramagopalan SV Goldacre R Skingsley A Conlon C Goldacre MJ. Associations between selected immune-mediated diseases and tuberculosis: record-linkage studies. BMC Med. (2013) 11:97. doi: 10.1186/1741-7015-11-97, PMID: 23557090 PMC3616814

[90] Balakrishnan C Mangat G Mittal G Joshi VR. Tuberculosis in patients with systemic lupus erythematosus. J Assoc Phys India. (1998) 46:682–3. 11229272

[91] Hou C-L Tsai Y-C Chen L-C Huang J-L. Tuberculosis infection in patients with systemic lupus erythematosus: pulmonary and extra-pulmonary infection compared. Clin Rheumatol. (2008) 27:557–63. doi: 10.1007/s10067-007-0741-8, PMID: 17940720

[92] Lao M Chen D Wu X Chen H Qiu Q Yang X. Active tuberculosis in patients with systemic lupus erythematosus from Southern China: a retrospective study. Clin Rheumatol. (2019) 38:535–43. doi: 10.1007/s10067-018-4303-z, PMID: 30244432

[93] Ahmmed MF Islam MN Ferdous S Azad AK Ferdous N. Tuberculosis in systemic lupus erythematosus patients. Mymensingh Med J. (2019) 28:797–807., PMID: 31599243

[94] González-Naranjo LA Coral-Enríquez JA Restrepo-Escobar M Muñoz-Vahos CH Jaramillo-Arroyave D Vanegas-García AL. Factors associated with active tuberculosis in Colombian patients with systemic lupus erythematosus: a case-control study. Clin Rheumatol. (2021) 40:181–91. doi: 10.1007/s10067-020-05225-x, PMID: 32529420

[95] Arenas Miras Mdel M Hidalgo-Tenorio C Jimenez-Gamiz P Jimenez-Alonso J. Diagnosis of latent tuberculosis in patients with systemic lupus erythematosus: T.SPOT.TB versus tuberculin skin test. BioMed Res Int. (2014) 2014:291031. doi: 10.1155/2014/291031, PMID: 25009813 PMC4058455

[96] Domínguez J Latorre I. Aplicación y utilidad actual de las técnicas de interferón-γ en el diagnóstico de la tuberculosis. Enfermedades Infecciosas y Microbiol Clínica. (2015) 33:15–9. doi: 10.1016/S0213-005X(15)30010-0, PMID: 26320991

[97] Hussein DAE-M Habeeb RAE-M El-Azizi NO Salah El-Deen NNM Morad CS Hawwash AM. Mycobacterium tuberculosis infection in systemic lupus erythematosus patients. Egyptian Rheumatol. (2017) 39:227–31. doi: 10.1016/j.ejr.2017.04.005

[98] Takeda N Nojima T Terao C Yukawa N Kawabata D Ohmura K. Interferon-gamma release assay for diagnosing Mycobacterium tuberculosis infections in patients with systemic lupus erythematosus. Lupus. (2011) 20:792–800. doi: 10.1177/0961203310397966, PMID: 21562022

[99] Cho H Kim YW Suh CH Jung JY Um YJ Jung JH. Concordance between the tuberculin skin test and interferon gamma release assay (IGRA) for diagnosing latent tuberculosis infection in patients with systemic lupus erythematosus and patient characteristics associated with an indeterminate IGRA. Lupus. (2016) 25:1341–8. doi: 10.1177/0961203316639381, PMID: 26985011

[100] Fragoulis GE Nikiphorou E Dey M Zhao SS Courvoisier DS Arnaud L. 2022 EULAR recommendations for screening and prophylaxis of chronic and opportunistic infections in adults with autoimmune inflammatory rheumatic diseases. Ann Rheum Dis. (2023) 82:742–53. doi: 10.1136/ard-2022-223335, PMID: 36328476

[101] Dlugovitzky D Torres-Morales A Rateni L Farroni MA Largacha C Molteni O. Circulating profile of Th1 and Th2 cytokines in tuberculosis patients with different degrees of pulmonary involvement. FEMS Immunol Med Microbiol. (1997) 18:203–7. doi: 10.1111/j.1574-695X.1997.tb01046.x, PMID: 9271171

[102] Aldakheel FM Alshanqiti MA Alduraywish SA Alshammary AF Dabwan KH Syed R. Clinical assessment of cytokine profiles and haematological parameters in patients with systemic lupus erythematosus: A cross-sectional study from Saudi Arabia. Front Biosci (Landmark Ed). (2023) 28:358. doi: 10.31083/j.fbl2812358, PMID: 38179775

[103] Sampath P Rajamanickam A Thiruvengadam K Natarajan AP Hissar S Dhanapal M. Cytokine upsurge among drug-resistant tuberculosis endorse the signatures of hyper inflammation and disease severity. Sci Rep. (2023) 13:785. doi: 10.1038/s41598-023-27895-8, PMID: 36646786 PMC9842614

[104] Cho H Kim YW Suh CH Jung JY Um YJ Jung JH. Concordance between the tuberculin skin test and interferon gamma release assay (IGRA) for diagnosing latent tuberculosis infection in patients with systemic lupus erythematosus and patient characteristics associated with an indeterminate IGRA. Lupus. (2016) 25:1341–8. doi: 10.1177/0961203316639381, PMID: 26985011

[105] Winslow GM Cooper A Reiley W Chatterjee M Woodland DL. Early T-cell responses in tuberculosis immunity. Immunol Rev. (2008) 225:284–99. doi: 10.1111/j.1600-065X.2008.00693.x, PMID: 18837789 PMC3827678

[106] Theofilopoulos AN Koundouris S Kono DH Lawson BR. The role of IFN-gamma in systemic lupus erythematosus: a challenge to the Th1/Th2 paradigm in autoimmunity. Arthritis Res Ther. (2001) 3:136. doi: 10.1186/ar290, PMID: 11299053 PMC128889

[107] Ehlers S. Role of tumour necrosis factor (TNF) in host defence against tuberculosis: implications for immunotherapies targeting TNF. Ann Rheum Dis. (2003) 62:ii37–42. doi: 10.1136/ard.62.suppl_2.ii37, PMID: 14532147 PMC1766754

[108] Dorhoi A Kaufmann SH. Tumor necrosis factor alpha in mycobacterial infection. Semin Immunol. (2014) 26:203–9. doi: 10.1016/j.smim.2014.04.003, PMID: 24819298

[109] Aringer M Feierl E Steiner G Stummvoll GH Höfler E Steiner CW. Increased bioactive TNF in human systemic lupus erythematosus: associations with cell death. Lupus. (2002) 11:102–8. doi: 10.1191/0961203302lu160oa, PMID: 11958572

[110] Kollias G Kontoyiannis D Douni E Kassiotis G. The role of TNF/TNFR in organ-specific and systemic autoimmunity: implications for the design of optimizedAnti-TNF’Therapies. Curr Dir Autoimmun. (2002) 5:30–50. doi: 10.1016/S1359-6101(02)00019-9, PMID: 11826759

[111] Aujla SJ Dubin PJ Kolls JK. Th17 cells and mucosal host defense. Semin Immunol. (2007) 19:377–82. doi: 10.1016/j.smim.2007.10.009, PMID: 18054248 PMC2293278

[112] Piergallini TJ Turner J. Tuberculosis in the elderly: Why inflammation matters. Exp Gerontol. (2018) 105:32–9. doi: 10.1016/j.exger.2017.12.021, PMID: 29287772 PMC5967410

[113] Turner J Orme IM. The expression of early resistance to an infection with Mycobacterium tuberculosis by old mice is dependent on IFN type II (IFN-gamma) but not IFN type I. Mech Ageing Dev. (2004) 125:1–9. doi: 10.1016/j.mad.2003.09.002, PMID: 14706232

[114] Turner J Frank AA Orme IM. Old mice express a transient early resistance to pulmonary tuberculosis that is mediated by CD8 T cells. Infect Immun. (2002) 70:4628–37. doi: 10.1128/IAI.70.8.4628-4637.2002, PMID: 12117976 PMC128196

[115] Cooper AM Callahan JE Griffin JP Roberts AD Orme IM. Old mice are able to control low-dose aerogenic infections with Mycobacterium tuberculosis. Infect Immun. (1995) 63:3259–65. doi: 10.1128/iai.63.9.3259-3265.1995, PMID: 7642254 PMC173449

[116] Canan CH Gokhale NS Carruthers B Lafuse WP Schlesinger LS Torrelles JB. Characterization of lung inflammation and its impact on macrophage function in aging. J Leukoc Biol. (2014) 96:473–80. doi: 10.1189/jlb.4A0214-093RR, PMID: 24935957 PMC4632167

[117] Vesosky B Rottinghaus EK Davis C Turner J. CD8 T Cells in old mice contribute to the innate immune response to Mycobacterium tuberculosis via interleukin-12p70-dependent and antigen-independent production of gamma interferon. Infect Immun. (2009) 77:3355–63. doi: 10.1128/IAI.00295-09, PMID: 19470747 PMC2715662

[118] Vesosky B Flaherty DK Turner J. Th1 cytokines facilitate CD8-T-cell-mediated early resistance to infection with Mycobacterium tuberculosis in old mice. Infect Immun. (2006) 74:3314–24. doi: 10.1128/IAI.01475-05, PMID: 16714559 PMC1479270

[119] Vesosky B Turner J. The influence of age on immunity to infection with Mycobacterium tuberculosis. Immunol Rev. (2005) 205:229–43. doi: 10.1111/j.0105-2896.2005.00257.x, PMID: 15882357

[120] Satti I Wittenberg RE Li S Harris SA Tanner R Cizmeci D. Inflammation and immune activation are associated with risk of Mycobacterium tuberculosis infection in BCG-vaccinated infants. Nat Commun. (2022) 13:6594. doi: 10.1038/s41467-022-34061-7, PMID: 36329009 PMC9632577

[121] Fletcher HA Snowden MA Landry B Rida W Satti I Harris SA. T-cell activation is an immune correlate of risk in BCG vaccinated infants. Nat Commun. (2016) 7:11290. doi: 10.1038/ncomms11290, PMID: 27068708 PMC4832066

[122] Müller J Tanner R Matsumiya M Snowden MA Landry B Satti I. Cytomegalovirus infection is a risk factor for tuberculosis disease in infants. JCI Insight. (2019) 4. doi: 10.1172/jci.insight.130090, PMID: 31697647 PMC6962026

[123] Amital-Teplizki H Avinoach I Coates A Kooperman O Blank M Shoenfeld Y. Binding of monoclonal anti-DNA and anti-TB glycolipids to brain tissue. Autoimmunity. (1989) 4:277–87. doi: 10.3109/08916938909014704, PMID: 2518831

[124] Sela O El-Roeiy A Isenberg D Kennedy R Colaco C Pinkhas J. A common anti-DNA idiotype in sera of patients with active pulmonary tuberculosis. Arthritis Rheumatism. (1987) 30:50–6. doi: 10.1002/art.1780300107, PMID: 3814197

[125] Shoenfeld Y Vilner Y Coates AR Rauch J Lavie G Shaul D. Monoclonal anti-tuberculosis antibodies react with DNA, and monoclonal anti-DNA autoantibodies react with Mycobacterium tuberculosis. Clin Exp Immunol. (1986) 66:255–61. PMC1542527 3102132

[126] Al-Arbi KMS Magula NP Mody GM. Tuberculosis remains a major burden in systemic lupus erythematosus patients in Durban, South Africa. Front Med (Lausanne). (2023) 10:1118390. doi: 10.3389/fmed.2023.1118390, PMID: 36936236 PMC10014752

[127] Beydon M Nicaise-Roland P Mageau A Farkh C Daugas E Descamps V. Autoantibodies against IFNα in patients with systemic lupus erythematosus and susceptibility for infection: a retrospective case-control study. Sci Rep. (2022) 12:11244. doi: 10.1038/s41598-022-15508-9, PMID: 35788140 PMC9253327

[128] Ahamada MM Jia Y Wu X. Macrophage polarization and plasticity in systemic lupus erythematosus. Front Immunol. (2021) 12:734008. doi: 10.3389/fimmu.2021.734008, PMID: 34987500 PMC8721097

[129] Ghorbaninezhad F Leone P Alemohammad H Najafzadeh B Nourbakhsh NS Prete M. Tumor necrosis factor−α in systemic lupus erythematosus: Structure, function and therapeutic implications (Review). Int J Mol Med. (2022) 49. doi: 10.3892/ijmm.2022.5098, PMID: 35137914

[130] Youssef J Novosad SA Winthrop KL. Infection risk and safety of corticosteroid use. Rheum Dis Clin North Am. (2016) 42:157–76, ix-x. doi: 10.1016/j.rdc.2015.08.004, PMID: 26611557 PMC4751577

[131] Li PH Lau C-S. Efficacy and safety of vaccinations in systemic lupus erythematosus. J Clin Rheumatol Immunol. (2020) 20:35–41. doi: 10.1142/S2661341720300037

[132] Harpaz R Ortega-Sanchez IR Seward JF. Prevention of herpes zoster: recommendations of the Advisory Committee on Immunization Practices (ACIP). Morbidity Mortality Weekly Report: Recommend Rep. (2008) 57:1–30. 18528318

[133] Shoenfeld Y Aron-Maor A Tanai A Ehrenfeld M. BCG and autoimmunity: another two-edged sword. J Autoimmun. (2001) 16:235–40. doi: 10.1006/jaut.2000.0494, PMID: 11334488

[134] Valentini D Rao M Rane L Rahman S Axelsson-Robertson R Heuchel R. Peptide microarray-based characterization of antibody responses to host proteins after bacille Calmette-Guérin vaccination. Int J Infect Dis. (2017) 56:140–54. doi: 10.1016/j.ijid.2017.01.027, PMID: 28161459

[135] Baxter AG Horsfall AC Healey D Ozegbe P Day S Williams DG. Mycobacteria precipitate an SLE-like syndrome in diabetes-prone NOD mice. Immunology. (1994) 83:227–31., PMID: 7835939 PMC1414944

[136] Ropper AH Victor M. Influenza vaccination and the Guillain–Barre syndrome. Mass Med Soc. (1998) 339:1845–6. doi: 10.1056/NEJM199812173392509, PMID: 9854122

[137] Ristori G Romano S Cannoni S Visconti A Tinelli E Mendozzi L. Effects of Bacille Calmette-Guerin after the first demyelinating event in the CNS. Neurology. (2013) 82:10–1212. doi: 10.1212/01.wnl.0000438216.93319.ab, PMID: 24306002 PMC3873620

[138] Faustman DL Wang L Okubo Y Burger D Ban L Man G. Proof-of-concept, randomized, controlled clinical trial of Bacillus-Calmette-Guerin for treatment of long-term type 1 diabetes. PloS One. (2012) 7:e41756. doi: 10.1371/journal.pone.0041756, PMID: 22905105 PMC3414482

[139] Keefe RC Takahashi H Tran L Nelson K Ng N Kühtreiber WM. BCG therapy is associated with long-term, durable induction of Treg signature genes by epigenetic modulation. Sci Rep. (2021) 11:14933. doi: 10.1038/s41598-021-94529-2, PMID: 34294806 PMC8298580

[140] Yu CB Jiang R Yy N Shui W Gao Q Zhang CR. Therapeutic action research of Bacille Calmette Guerin (BCG) on systemic lupus erythematosus mouse model. J Immunol Techniques Infect Dis. (2016) 5.

[141] Canete R Picazo E Guzman R Eslava A Guzman A Villasor R. Beneficial role of BCG vaccination in the management of systemic lupus erythematosus: a report of three cases. Phil J Internal Med. (1983) 329–34.

[142] Tait DR Hatherill M van der Meeren O Ginsberg AM Van Brakel E Salaun B. Final analysis of a trial of M72/AS01(E) vaccine to prevent tuberculosis. N Engl J Med. (2019) 381:2429–39. doi: 10.1056/NEJMoa1909953, PMID: 31661198

[143] Spertini F Audran R Chakour R Karoui O Steiner-Monard V Thierry AC. Safety of human immunisation with a live-attenuated Mycobacterium tuberculosis vaccine: a randomised, double-blind, controlled phase I trial. Lancet Respir Med. (2015) 3:953–62. doi: 10.1016/S2213-2600(15)00435-X, PMID: 26598141

[144] Kumarasamy N Poongulali S Beulah FE Akite EJ Ayuk LN Bollaerts A. Long-term safety and immunogenicity of the M72/AS01E candidate tuberculosis vaccine in HIV-positive and -negative Indian adults: Results from a phase II randomized controlled trial. Medicine. (2018) 97:e13120. doi: 10.1097/MD.0000000000013120, PMID: 30407329 PMC6250513

[145] Soybilgic A Onel KB Utset T Alexander K Wagner-Weiner L. Safety and immunogenicity of the quadrivalent HPV vaccine in female Systemic Lupus Erythematosus patients aged 12 to 26 years. Pediatr Rheumatol Online J. (2013) 11:29. doi: 10.1186/1546-0096-11-29, PMID: 23924237 PMC3751269

